# An Immune–Magnetophoretic Device for the Selective and Precise Enrichment of Circulating Tumor Cells from Whole Blood

**DOI:** 10.3390/mi11060560

**Published:** 2020-05-30

**Authors:** Chaithanya Chelakkot, Jiyeon Ryu, Mi Young Kim, Jin-Soo Kim, Dohyeong Kim, Juhyun Hwang, Sung Hoon Park, Seok Bum Ko, Jeong Won Park, Moon Youn Jung, Ryong Nam Kim, Kyoung Song, Yu Jin Kim, Yoon-La Choi, Hun Seok Lee, Young Kee Shin

**Affiliations:** 1 Technical Research Center, Genobio Corp., Seoul 08394, Korea; lakshmi@genobio.co.kr (C.C.); jyryu1120@gmail.com (J.R.); dohyeongkim@genobio.co.kr (D.K.); jhhwang@genobio.co.kr (J.H.); shpark@genobio.co.kr (S.H.P.); koseokbum@gmail.com (S.B.K.); 2 Department of Internal Medicine, Seoul National University Boramae Medical Center, Seoul 07061, Korea; moung9805@naver.com (M.Y.K.); gistmd@gmail.com (J.-S.K.); 3 IT Convergence Technology Research Laboratory, Electronic and Telecommunications Research Institute, Daejon 34129, Korea; jwp0422@etri.re.kr (J.W.P.); j83637218@gmail.com (M.Y.J.); 4 Bio-MAX/N-Bio, Seoul National University, Seoul 08826, Korea; ryongnamkim@gmail.com; 5 The Center for Companion Diagnostics, LOGONE Bio Convergence Research Foundation, Seoul 08394, Korea; sk17@logonebio.org (K.S.); yujinkim@logonebio.org (Y.J.K.); 6 Laboratory of Cancer Genomics and Molecular Pathology, Samsung Medical Center, Sungkyunkwan University School of Medicine, Seoul 08394, Korea; yla.choi@samsung.com; 7 Department of Pathology and Translational Genomics, Samsung Medical Center, Sungkyunkwan University School of Medicine, Seoul 06351, Korea; 8 Laboratory of Molecular Pathology and Cancer Genomics, College of Pharmacy and Research Institute of Pharmaceutical Science, Seoul National University, Seoul 08826, Korea; 9 Department of Molecular Medicine and Biopharmaceutical Sciences, Graduate School of Convergence Science and Technology, Seoul National University, Seoul 08826, Korea; 10 The Center for Anti-Cancer Companion Diagnostics, Bio-MAX/N-Bio, Seoul National University, Seoul 08826, Korea

**Keywords:** circulating tumor cells, liquid biopsy, epithelial cell adhesion molecule (EpCAM), MET, biomarker

## Abstract

Here, we validated the clinical utility of our previously developed microfluidic device, GenoCTC, which is based on bottom magnetophoresis, for the isolation of circulating tumor cells (CTCs) from patient whole blood. GenoCTC allowed 90% purity, 77% separation rate, and 80% recovery of circulating tumor cells at a 90 μL/min flow rate when tested on blood spiked with epithelial cell adhesion molecule (EpCAM)-positive Michigan Cancer Foundation-7 (MCF7) cells. Clinical studies were performed using blood samples from non-small cell lung cancer (NSCLC) patients. Varying numbers (2 to 114) of CTCs were found in each NSCLC patient, and serial assessment of CTCs showed that the CTC count correlated with the clinical progression of the disease. The applicability of GenoCTC to different cell surface biomarkers was also validated in a cholangiocarcinoma patient using anti-EPCAM, anti-vimentin, or anti-tyrosine protein kinase MET (c-MET) antibodies. After EPCAM-, vimentin-, or c-MET-positive cells were isolated, CTCs were identified and enumerated by immunocytochemistry using anti-cytokeratin 18 (CK18) and anti-CD45 antibodies. Furthermore, we checked the protein expression of PDL1 and c-MET in CTCs. A study in a cholangiocarcinoma patient showed that the number of CTCs varied depending on the biomarker used, indicating the importance of using multiple biomarkers for CTC isolation and enumeration.

## 1. Introduction

Liquid biopsies allow the sampling and analysis of cancer-derived elements present in body fluids, primarily blood, of cancer patients, such as circulating or disseminating tumor cells (CTCs/DTCs), circulating tumor DNA (ctDNA), and exosomes, which are proving to be surrogate biomarkers for cancer detection and progression [1,2,3]. These sources can be used in place of conventional tissue biopsies, which do not perform consistently in real-time and provide limited information because of their invasive nature and difficulty in acquisition. Furthermore, liquid biopsy samples can be obtained more frequently to provide a snapshot of the disease at critical time points [2,4,5]. This is tremendously important because the acquired data will provide temporal information regarding disease progression and early evidence of cancer metastasis, recurrence, and treatment resistance during the therapeutic period. Though the United States, food, and drug administration (US FDA) has approved the use of CellSearch^®^ CTC as a useful prognostic method for lung cancer, breast cancer, and prostate cancer, the clinical implementation of CTCs in cancer evaluation is not widespread. The development of technologies that enable accurate and high-throughput sorting of CTCs, well-suited for clinical and industrial use, is imperative to fully exploit the potential of CTCs.

Since the prognostic and diagnostic potential of CTCs was identified, numerous methods have been employed to obtain CTC-enriched samples [6,7,8,9]. CellSearch^®^ employs an anti-EPCAM antibody for CTC detection and is the most widely used device in translational research [10,11,12]. In addition, other methods similar to CellSearch^®^, such as a technique based on the use of anti-epithelial cell adhesion molecule (EpCAM)-coated micro-posts or micro-channels, have been realized to capture CTCs effectively [13,14]. An alternative strategy for CTC enrichment takes advantage of the differences in physical properties, including size, density, and deformability, between CTCs and leukocytes [7]. Microfiltration is the best-developed method among these techniques and separates CTCs from leukocytes on the basis of the larger size [15,16,17]. This allows the collection of CTCs regardless of specific markers, resulting in high recovery. However, such technologies fail to detect small-sized CTCs ranging from 6 µm to 8 µm or those with a small nucleus [18], which are similar in size to leukocytes [19,20]. Moreover, such methods frequently result in extremely low-purity samples because of white blood cells (WBC) contamination, generating unreliable data in the subsequent molecular characterization. Indeed, highly specialized technologies are still needed to achieve considerable advances in CTC isolation because of rarity and purity issues.

Here, we developed GenoCTC, a novel CTC-isolating device, which relies on microfluidics and lateral magnetophoresis for the efficient separation of CTCs, which is stimulated by a magnetic field gradient-based force. The bottom magnetophoresis system selectively and precisely isolates CTCs using specific antibody-coated magnetic microbeads, whereas the top microfluidic platform controls the flow rate of the sample [21]. We optimized and validated GenoCTC, by spiking healthy blood samples with cancer cells and found a notably high recovery rate of 80%, a separation rate of 77%, and a purity of 90%. Clinical feasibility was investigated by analyzing whole blood specimens from non-small cell lung cancer (NSCLC) patients and cholangiocarcinoma patient, using the epithelial marker EPCAM and the epithelial–mesenchymal transition (EMT) biomarkers vimentin and MET. In addition to enumerating CTCs, we investigated potent cancer biomarkers such as PDL1 and MET in the isolated CTCs. Overall, the results of our study indicate that GenoCTC is a reliable and sensitive device for CTC isolation from different cancers. The device can also be used to isolate the target cells of interest using different cell surface biomarkers. Moreover, downstream molecular characterization of the isolated CTCs is also possible, thereby making GenoCTC a promising in vitro diagnostic device to improve cancer diagnosis.

## 2. Materials and Methods

### 2.1. Device Design and Microchip Fabrication

**Figure 1:**
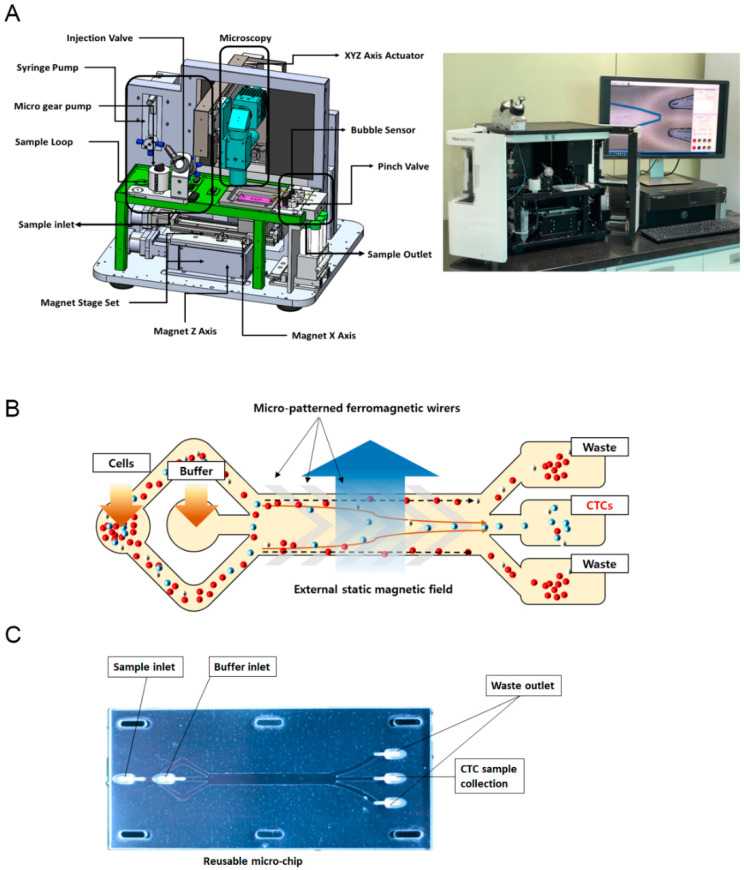
Schematic representation of GenoCTC and GenoChip: (**A**) Schematic diagram showing the microfluidic system of GenoCTC. All components that produce the microfluidic force and magnetic field for isolating circulating tumor cells (CTCs) are indicated. (**B**) Graphical image of GenoChip. CTCs are isolated by a magnetic gradient-based force using lateral magnetophoresis. The microfabricated ferromagnetic stripes generate a magnetic trap so that cells bound to a sufficient number of magnetic beads are trapped over the magnetic stripes and move along the stripe direction, rather than parallel to the fluid flow. (**C**) Actual image of the updated disposable chip.

GenoCTC is a device for the collection of liquid biopsies which drives a microfluidic control system (Figure 1A). A micro gear pump and a syringe pump enable to transfer the blood sample and the buffer to the inner part of a microchip and control the flow rate, while reducing the flow fluctuation in the fluid. The magnet stage is placed underneath the microchip and generates a magnetic field, which allows the isolation of the targeted cells bound to immunomagnetic microbeads. The motion stage is equipped with an optic system using a 4X lens and an XYZ axis stepper motor for tracing the inside of the microchip and observe the whole process of cell isolation. The workstation connected to the device enables to monitor the process in real time and manage the system, such as device initialization, flow rate control, and video recording, as well as the injection of the buffer. The sample to be analyzed is injected manually through an injection valve using a Hamilton syringe.

The working principle of the microchip was reported in our previous study [21]. Briefly, the Geno microchip consists of a bottom glass substrate, fabricated with inlaid v-patterned 60 µm-thick and 100 µm-wide electroporated nickel cobalt (Ni–Co) ferromagnetic wires which generate the magnetophoretic field, and of a top polydimethylsiloxane (PDMS; Sylgard 184, Dow Corning, Midland, MI, USA) substrate consisting of microchannels that control the microfluidic flow (Figure S1A). Each microchannel is 3 mm in width, has a height of 50 mm, and allows the flow of samples and buffer. The top PDMS substrate is bonded to an SU-8 film-coated bottom wafer using oxygen plasma treatment followed by coating with 5% 3-aminopropyltrmethoxysilane (APTES; Sigma Aldrich, St. Louis, MO, USA) in 95% ethanol, which ensures permanent sealing. We also newly designed a disposable microchip fabricated with polymethyl methacrylate (PMMA, LOTTE Chemical. Corp., Seoul, Korea) containing microchannels bonded to a polyester (PET) film (SKC Co., Ltd., Suwon, Korea) using a double-sided tape (3M, Maplewood, MN). Such disposable microchip is assembled above the bottom glass wafer integrated with the ferromagnetic wires and can be replaced in each test of clinical samples, while the bottom wafer is reusable.

### 2.2. Preparation of Antibody-Coated Magnetic Microbeads

Magnetic microbead-conjugated antibodies for CTC isolation were prepared in phosphate-buffered saline (PBS; Gibco, Thermo Fisher Scientific, Inc., Waltham, MA, USA) by incubation of biotinylated anti-human EPCAM (eBioscience, Thermo Fisher Scientific, Inc.), biotinylated anti-human vimentin (R&D systems, Biotechne., Minneapolis, MN, USA), or biotinylated anti-human MET (eBioscience, Thermo Fisher Scientific, Inc.) with streptavidin-coated magnetic beads (Size 1 µm) (Dynabead, Invitrogen, Carlsbad, CA, USA) for 1 h at room temperature. For the preparation, 1 mL of antibody–magnetic bead conjugate, 35 µL of a 0.5 µg/mL antibody solution, and 70 µL of magnetic beads at the concentration of 10 mg/mL (7–10 × 10^9^ beads/mL) were mixed with 895 µL of PBS.

### 2.3. Cell Culture and Blood Sample Collection

The human breast cancer cell lines MCF7, MDAMB453, HCC1187, and MDAMB231 and the human NSCLC cell lines NCIH1755 and HCC44 were obtained from Korean Cell Line Bank (KCLB; Seoul, Korea). The cell lines were maintained in RPMI 1640 (Thermo Fisher Scientific, Inc.) supplemented with 10% fetal bovine serum (FBS; Thermo Fisher Scientific, Inc.) and penicillin (100 U/mL)/streptomycin (100 μg/mL) (Hyclone, Thermo Fisher Scientific, Inc.) in 100 mm culture dishes (Corning Inc., Corning, NY, USA) at 37 °C in a 5% CO_2_ atmosphere. The cells were harvested at 80% confluence and dissociated using Accutase^TM^ (Stemcell Technologies, Vancouver, BC, Canada) in all experiments.

For the spiking test, normal blood samples were purchased from Innovative Research (Innovative research, Peary Count, Novi, MI, USA). For the clinical study, whole blood samples from 30 healthy volunteers were purchased from Innovative Research (Innovative research, Peary Count, Novi, MI, USA). Blood samples from 10 patients with advanced or metastatic NSCLC were collected from Seoul National University Boramae Medical Center (Korea) under the Institutional Review Board (IRB)-approved protocol (IRB number: 16-2014-63). All studies were performed after obtaining informed consent from the patients. The samples were collected in Vacutainer™ tubes (BD Biosciences, Franklin Lakes, NJ, USA) containing ethylene diamine tetraacetic acid (EDTA) as an anticoagulant and processed within three hours.

For the cholangiocarcinoma study, a blood sample was collected from a cholangiocarcinoma patient at Seoul National University Bundang Hospital, Seoul (blood samples from leftover de-identifiable samples, not requiring IRB approval, were used). The samples were collected in Vacutainer™ tubes (BD Biosciences, Franklin Lakes, NJ, USA) containing EDTA as an anticoagulant and processed within three hours.

### 2.4. Sample Preparation

For the GenoCTC optimization tests and analytical validation, 4 × 10^4^ cancer cells were suspended in 100 μL PBS (Thermo Fisher Scientific, Inc.) and incubated with 29 μL of anti-human EPCAM-coated magnetic microbeads for 1 h at room temperature. The sample for the spiking experiment was prepared by spiking 2 × 10^3^ MCF7 in 100–733 μL of healthy whole blood. MCF7 cells were stained with Hoechst^®^ (Thermo Fisher Scientific, Inc.) prior to spiking to distinguish them from blood cells. Then, 167 μL of anti-human EPCAM-coated magnetic microbeads was added to the blood sample and incubated for an hour at room temperature. For the isolation of cells from the clinical samples, 3.5 mL of whole blood was mixed with 525 μL of PBS and incubated with 175 μL of anti-human EPCAM-/anti-human vimentin-/or anti-human MET-coated magnetic microbeads for 1 h at room temperature.

### 2.5. Immunocytochemistry

Isolated EPCAM-, vimentin-, and MET-positive cells were centrifuged with CytoSpin (Thermo Fisher Scientific, Inc.) at 40 *g* for 5 min, and fixed in in 4% paraformaldehyde (Sigma Aldrich) for 10 min. the fixed cells were washed twice using PBS (Thermo Fisher Scientific, Inc.) with 1% bovine serum albumin (BSA; Sigma Aldrich) and permeabilized with 0.2% Tween 20 (Sigma Aldrich) for 10 min. Then, the permeabilized cells were blocked with 2% BSA for 30 min under humid conditions. The samples were incubated for 2 h with the antibodies in a humid box at room temperature, followed by three washes with PBS. The following antibodies were used: Alexa 488-conjugated anti- cytokeratin 18 (CK18) (eBioscience, Thermo Fisher Scientific, Inc.), Alexa 594-conjugated anti-pan CK (BioLegend, Thermo Fisher Scientific, Inc.), Cy3/Alexa594-conjugated anti-CD45 (eBioscience, Thermo Fisher Scientific, Inc.), Alexa 488-conjugated anti-MET (eBioscience, Thermo Fisher Scientific, Inc.), and Alexa 488-conjugated anti-PDL1 (Spring Biosciences, Pleasanton, CA, USA). For detecting PDL1 signal, Alexa flour 488-conjugated anti-rabbit (Thermo Fisher Scientific, Inc.) was also used, and incubation was carried out for 1 h. Finally, the cells were mounted with DAPI (Vector Laboratories, Inc, Burlingame, California, USA) for nuclei staining. The images were captured using a Nikon Eclipse Ni microscope equipped with an Infinity3 camera (Nikon Eclipse Inc., Tokyo, Japan)

### 2.6. Flow Cytometry

In total, 2 × 10^5^ cells were prepared and stained with anti-EPCAM antibodies (1:40, FITC-conjugated, eBioscience, Thermo Fisher Scientific, Inc.) for 1 h at room temperature in the dark. Next, the cells were washed with 2% FBS in PBS and fixed in 4% paraformaldehyde for 20 min on ice in the dark. After washing one time, 1 × 10^4^ cells were quantified using a FACS Calibur flow cytometer (BD Biosciences). EPCAM expression level was analyzed by checking the shift of the peaks.

## 3. Results

### 3.1. GenoCTC Working Principle

We newly developed the GenoCTC microfluidic device to isolate EPCAM-positive cells, which are known to be putative CTCs, from whole blood using a microchip based on bottom magnetophoresis. A schematic design of the device and the microchip is shown in Figure 1A,B. The microchip is a fluidic control system that provides a microfluidic force and a magnetic field to isolate the target cells. Magnetic microbeads coated with anti-human EPCAM were used to selectively isolate EPCAM-positive cells under a magnetic field. We newly fabricated a disposable microchip, Genochip, using PMMA for microchannels and bonded it to the film with double-sided tape, obtaining an updated version of the assembled microchip [21]. The microchip (Figure 1C) consisted of two sample inlets and one buffer inlet and three outlets for the collection of the separated elements. An actual image of a blood sample being separated in the microchip is shown in supplementary Figure S1B. Specifically, the system has an approximately 8 cm chip integrated with microchannels and V-shaped Ni–Co ferromagnetic wires forming a magnetic field designed to guide and trap bead-bound cells along the direction of the wire, while the unbound cells, such as blood cells, move parallel to the fluid flow. An external magnetic field along with the ferromagnetic wires, which acts as a micromagnet, enhances the magnetic field locally along the path of the wire, making each stripe in the wire pattern work as a trap for cells bound to a sufficient number of magnetic beads. This allows improved capture and reduced contamination from WBCs during CTC isolation. The disposable microchip prevents cross-contamination issues between blood samples when independent experiments are conducted, while the inlaid ferromagnetic wires are reusable.

High capture efficiency, high throughput, and high isolation purity, while preserving cell viability, are critical factors to be considered in evaluating the performance of a CTC isolation system [22,23]. Unlike many studies in which the results were evaluated on the basis of separation or recovery rates according to an imprecise concept, our method enables the accurate calculation of the efficiency of cell isolation. A detailed evaluation of the performance of GenoCTC is presented below.

The recovery rate was calculated by comparing the total cell input with the total cell output.

(1) $$\mathrm{Recovery}\;(\%):\;\frac{Output\;cells\;\left(Waste+CTCs+WBC\;impurities\right)}{Total\;Input\;cells}\times 100$$

The separation rate was determined by calculating the proportion between untargeted cells—termed wastes—and EPCAM-positive cells, which were our target cells. The target cell compartment could contain some WBC contamination or impurities, which was taken into consideration while calculating the sample purity.

(2) $$\mathrm{Separation}\;\mathrm{rate}\;(\%):\;\frac{\;Targeted\;cells\;\left(CTCs+WBC\;impurities\right)}{Output\;cells\;\left(Waste+CTCs+WBC\;impurities\right)}\times 100$$

The purity measured putative EPCAM-positive cells within the targeted cells bound to the anti-EPCAM-coated magnetic beads.

(3) $$\mathrm{Purity}\;(\%):\;\frac{CTCs}{Targeted\;cells\;\left(CTCs+WBC\;impurities\right)}\times 100$$

### 3.2. Analytical Performanc of GenoCTC

**Figure 2:**
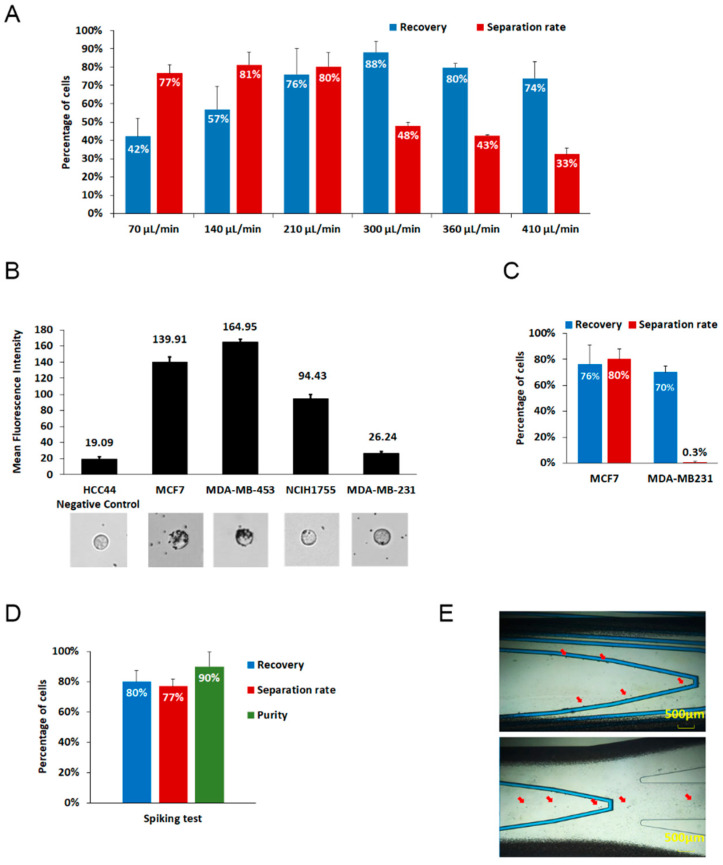
Performance of GenoCTC: (**A**) Microfluidic separation at various flow rates was performed using the EPCAM-positive cells MCF7 (breast cancer cell line), and the best performance was observed at a flow rate of 210 μL/min. (**B**) Mean fluorescence intensity of EPCAM-FITC for five cancer cell lines as quantified by flow cytometry. HCC44 and MDAMB231 cell lines were used as negative controls (no EpCAM expression). Images of anti-EPCAM-coated magnetic nanobeads binding to the cells are shown below the graph. (**C**) Separation and recovery rates for two different cell lines with varying EPCAM expression. EPCAM-positive MCF7 showed 76% recovery and 80% separation, whereas EPCAM-negative MDAMB231 showed about 70% recovery without any separation. (**D**) A total of 2 × 10^3^ magnetic nanobead-bound MCF7 cells were pre-labeled with Hoechst and suspended in a healthy donor’s whole blood. MCF7 cells were isolated at a flow rate of 90 μL/min and showed about 80% recovery, 72% separation, and 90% purity. (**E**) Images of the spiked sample during cell isolation. The images show the process of isolation of the spiked sample, and the red arrows in the images indicate bead-bound MCF7 cells, which were selectively isolated. Data are presented as the mean ± s.e.m of five experiments.

To optimize the isolating conditions, various flow rates were examined using the EPCAM-positive breast cancer cell line MCF7 in PBS buffer (Figure 2A). Using 4 × 10^5^ MCF7 cells, the best performance of GenoCTC was observed at the 210 μL/min flow rate, while slower flow rates (e.g., 70 μL/min and 140 μL/min) showed significant separation rates but lower recovery, while faster flow rates (300 μL/min, 360 μL/min, and 410 μL/min) retrieved more cells but with obviously lower separation efficiency. At higher flow rates, though the recovery rate increased, the separation rate dropped due to increased drag force relative to the magnetic force within the chip. The optimum separation rate using whole-blood specimens showed different outcomes compared to the previous results owing to the altered viscosity of the samples. The flow rate for the blood samples was optimized by adjusting the flow rate from the buffer-based test and it is considered appropriate to acquire CTCs with high purity rather than to retrieve a high number of cells that are contaminated with WBCs. While using whole-blood samples, the optimum flow rate was 90 μL/min.

We next investigated the effect of cellular EPCAM expression on the isolation efficiency of CTCs by using cell lines with different EPCAM expression. The EpCAM expression levels of the cell lines were verified by flow cytometry, and the EpCAM antibody–magnetic bead binding efficiency was tested. The six cancer cell lines examined showed different bead binding efficiency according to their EPCAM expression level determined by flow cytometry. Images of bead-bound cells are shown in Figure 2B (supporting data is shown in supplementary Figure S2). These results indicated the separation performance of GenoCTC when the cells were spiked into PBS; EPCAM-positive MCF7 cells were separated more efficiently than the other cells, and EPCAM-negative MDAMB231 cells were not isolated (Figure 2C). EpCAM expression was directly related to the number of magnetic beads that were bound to the cells. As expected, our results indicate that the efficiency of cell separation depended on the level of EPCAM expression, which reflects the performance of GenoCTC.

The major hurdle of the molecular analysis of CTCs is the background presence of nonspecific blood cells. Thus, the purity of CTCs is one of the critical points that must be considered when their genome is characterized. To examine the purity of GenoCTC, 2 × 10^3^ cells of MCF7 were spiked into whole blood from healthy donors. As shown in Figure 2D, the spiked samples allowed about 80% recovery, 77% separation rate, and 90% purity at 90 μL/min flow rate. Our recovery rate, separation rate, and purity of isolated CTC were relatively high, indicating that GenoCTC is a reliable and optimal device for the isolation of EPCAM-positive cells. As shown in Figure 2E, captured images of the isolation process of spiked samples revealed that bead-bound MCF7 cells followed the magnetic wires specifically, whereas untargeted blood cells flowed through both sides (Supplementary Videos S1 and S2). We further evaluated the efficiency of the GenoCTC system in isolating cells present in lower numbers, by spiking whole blood with serially diluted cells. Cell numbers ranging from 200 cells to 3 cells were suspended in whole blood, and the recovery rate and separation rate were calculated after processing the samples in the GenoCTC device. The recovery rate and separation rate showed an average of 77% and 73%, respectively, for cell numbers ranging from 200 to 6; however, they dropped to 43% and 40%, respectively, when the blood sample was spiked with three cells (Supplementary Figure S3).

### 3.3. Isolation and Enumeration of CTCs from NSCLC Patients

**Figure 3:**
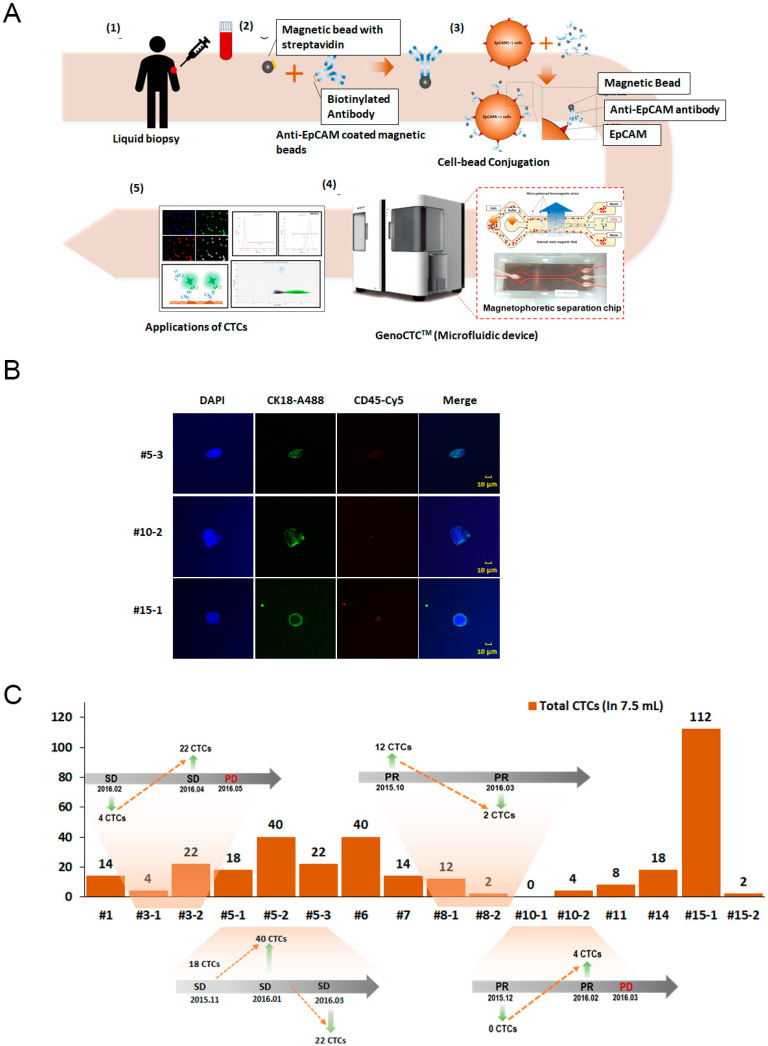
Isolation and enumeration of CTCs from non-small cell lung cancer (NSCLC) patients: (**A**) the detailed GenoCTC workflow using clinical samples is summarized as follows: (1) Whole blood is drawn from cancer patients. (2) The biotinylated anti-EPCAM antibody is conjugated with 1 μm streptavidin-coated magnetic nanobeads. (3) Anti-EPCAM antibody-coated beads and whole blood are incubated together, and the nanobeads will specifically bind onto EPCAM-positive cells. (4) EPCAM-positive cells are isolated using GenoCTC with a disposable GenoChip. (5) Molecular analysis is conducted in various ways using isolated EPCAM-positive cells. (**B**) Representative fluorescent images of isolated EPCAM-positive cells from three NSCLC patients are shown. Cells were immunostained with DAPI for nuclear staining (blue), an anti-cytokeratin 18 (CK18) antibody for CTCs (Alexa flour 488; Green), and an anti-CD45 antibody for leukocytes (Alexa 594; red). (**C**) EPCAM-positive cells isolated from the whole blood of 10 NSCLC patients were enumerated, and the number of cells observed varied between patients. EPCAM-positive cell counts ranged from 2 to 112 in 3.5 mL of whole blood, and the average count of the cells was 20.75, showing 95% confidence. The orange shaded area show the serial assessment of CTCs from those patients. The grey arrows indicate the clinical changes in the disease status determined by a physician. SD: stable disease, PR: partial response, PD: progressive disease. The orange arrows indicate the increase or decrease in CTC counts during the serial assessment.

The overall workflow of GenoCTC using clinical samples is as follows (Figure 3A). Whole blood was first acquired from a cancer patient and incubated for 1 h with anti-EPCAM antibody-conjugated magnetic beads. There is no need for pre-processing of the whole blood such as the isolation of peripheral blood mononuclear cells (PBMCs) or red blood cell lysis. The prepared sample was then injected into GenoCTC and GenoChip, after which EPCAM-positive cells isolated on this platform were collected for further molecular characterization. To analyze the performance of GenoCTC based on actual patient blood, 16 specimens of 3.5 mL blood from 10 advanced or metastatic NSCLC patients were assessed. CTC enumeration is well known to have prognostic value for the estimation of patients’ clinical outcome and enables monitoring the efficacy of treatments, as well as disease progression [24,25,26,27]. Among EPCAM-positive cells isolated through the GenoCTC platform, CTCs were identified by immunostaining with an anti-CK18 antibody directed towards a cancer epithelial cell marker and an anti-CD45 antibody recognizing a leukocyte marker (Figure 3B). DAPI+/CK18+/CD45− cells represent CTCs and were identified in 15 out of 16 patient samples, indicating approximately a 94% detection rate. Moreover, the number of CTCs isolated varied from 2 to 112 among patients (Figure 3C). In this cohort of clinical samples, the mean count of the CTCs was 20.75 cells, showing 95% confidence. Serial assessment of CTCs in 4 of these 15 patients showed that the CTC count was closely associated with the clinical progression of the disease. An increase in CTC count was observed in patients (patient #3 and patient #10) prior to clinical progression from a stable disease to progressive disease. Similarly, a drop in CTC count was observed in patients who showed partial response to therapy (patient #8) or did not show any progressive disease (patient #5). We also show that GenoCTC can efficiently isolate CTCs from a comparatively lower volume of blood (3.5 mL). The number of CTCs identified in each patient needs to be further investigated to determine if this level is clinically relevant to the disease status. Since only a small cohort of samples was analyzed in this study, the mean value and statistical significance may change when large-scale experiments are conducted. Furthermore, as shown in Figure 3B, each CTC showed different morphology. It is known that CTCs from different cancer types demonstrate morphologically broad heterogeneity, with CTCs of different cancers showing varying cellular or nuclear size, as well as irregular morphology. Such cytomorphologically abnormal CTCs have shown correlation with poor clinical outcome in metastatic breast, colorectal, and prostate cancer patients [18,19,20]. Recently, it was shown that the inclusion of large-sized cells, with abnormal nuclear-to-cytoplasmic ratios and irrespective of the expression of CTC-specific molecular markers, as CTCs might help in further uncovering the heterogeneity of these cells [28]. These studies indicate that the morphology of CTCs could be a promising biomarker beyond CTC enumeration and could potentially reduce the false negative results [9,18,29].

### 3.4. Programmed Death Ligand 1(PDL1) and Tyrosine Protein Kinase (MET) Expression on CTCs from Non-Small Cell Lung Cancer (NSCLC) Patients

Until recently, the focus of CTC research was on the development of novel technologies for the enrichment of CTCs. However, preservation of cell viability during the isolation procedure and downstream molecular characterization of isolated CTCs are gaining interest, as they would aid companion diagnostics in providing information to monitor disease progression and to design tailored and targeted therapies [30,31]. Studies have also shown that the expression of targetable biomarkers in CTCs, like PDL1 [32,33], human epidermal growth factor 2 (HER2) [34,35], and MET [36], sometimes correlates with that in the primary tumors while, in other cases, it shows significant changes.

**Figure 4:**
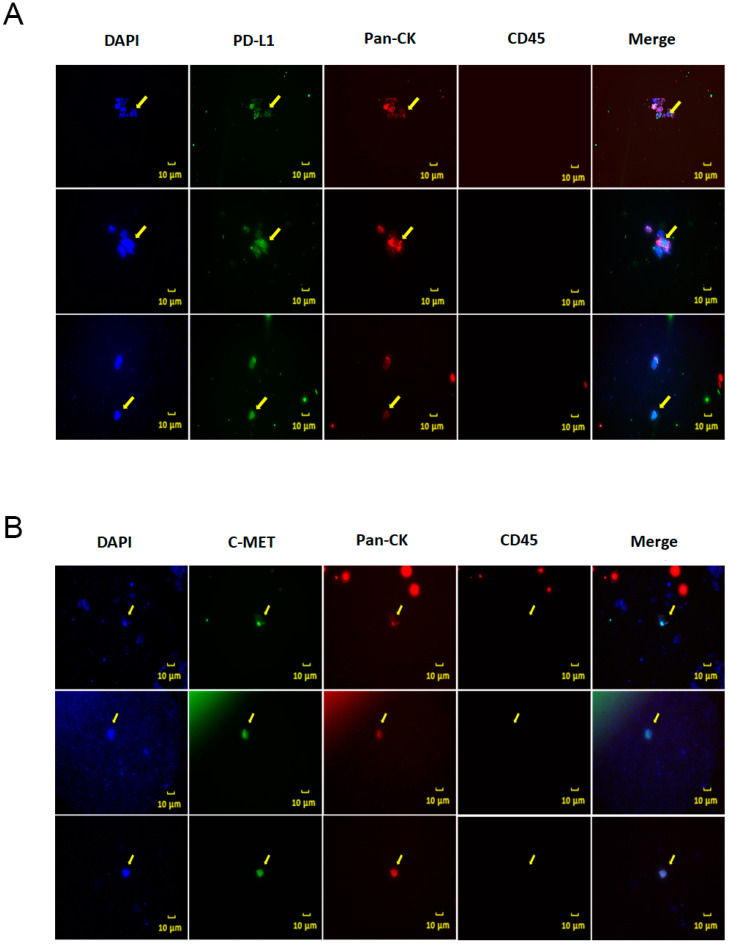
Characterization of isolated CTCs from NSCLC patients: (**A**) Immunofluorescence image showing EPCAM-positive cells isolated from an NSCLC patient (#5-3). EPCAM-positive cells were immunostained with DAPI for nuclear staining (blue), an anti-PDL1 antibody (Alexa flour 488; Green), and an anti-Pan CK antibody (Alexa flour 594; Orange red) for CTCs; an anti-CD45 antibody identified leukocytes (Cy3; Yellow). (**B**) EPCAM-positive cells isolated from an NSCLC patient (#15-1) were immunostained with DAPI for nuclear staining (blue), an anti-MET antibody (Alexa flour 488; Green), and an anti-Pan CK antibody (Alexa flour 594; Orange red) for CTCs; an anti-CD45 antibody was used for leukocytes (Cy3; Yellow). All microscopic images were obtained using a Nikon Eclipse Ni microscope equipped with an Infinity3 camera.

The identification of therapeutically targetable molecular markers on CTCs can thus help choose patients who would otherwise not be given a targeted therapy and would also not benefit from conventional therapies. Here, we investigated the levels of PDL1 and MET expression in isolated CTCs because they are two of the most promising therapeutic targets in cancer. As shown in Figure 4A, PDL1 expression was identified in EPCAM-positive CTCs isolated from a lung cancer patient. It is well known that aberrant expression of PDL1 occurs in various cancer types and is associated with poor survival. In addition, blocking PDL1 or its ligand PD1 has been reported to have a significant antitumor effect. In NSCLCs treatment, PDL1 immune checkpoint inhibitors have emerged as a new therapeutic approach. Previous studies have also suggested that PDL1-positive CTCs were a poor prognostic biomarker in several cancers and that they were highly associated with worse outcome in progression Free Survival (PFS) or overall survival (OS) [37,38,39]. Analysis of PDL1 expression in CTCs thus could provide valuable information for deciding therapeutic strategies. Moreover, MET expression was confirmed in CTCs isolated from a lung cancer patient (Figure 4B). MET is a receptor for hepatocyte growth factor (HGF), and its overexpression is related to the process of metastatic dissemination and drug resistance in several cancers, resulting in poor prognosis [40,41]. Furthermore, patients expressing MET-positive CTCs have been reported to show resistance to therapy. Therefore, MET-positive CTCs could be a potential predictive marker in deciding therapeutic strategies [36,42], also considering that therapies directed against MET have shown remarkable responses, specifically in NSCLC patients [43,44,45,46]. Further studies are essential to understand the role of MET-positive CTCs in overall survival as well as response to therapy of these patients.

### 3.5. Enumeration and Analysis of CTCs from a Cholangiocarcinoma Patient Using Epithelial and Non-Epithelial Markers 

**Figure 5:**
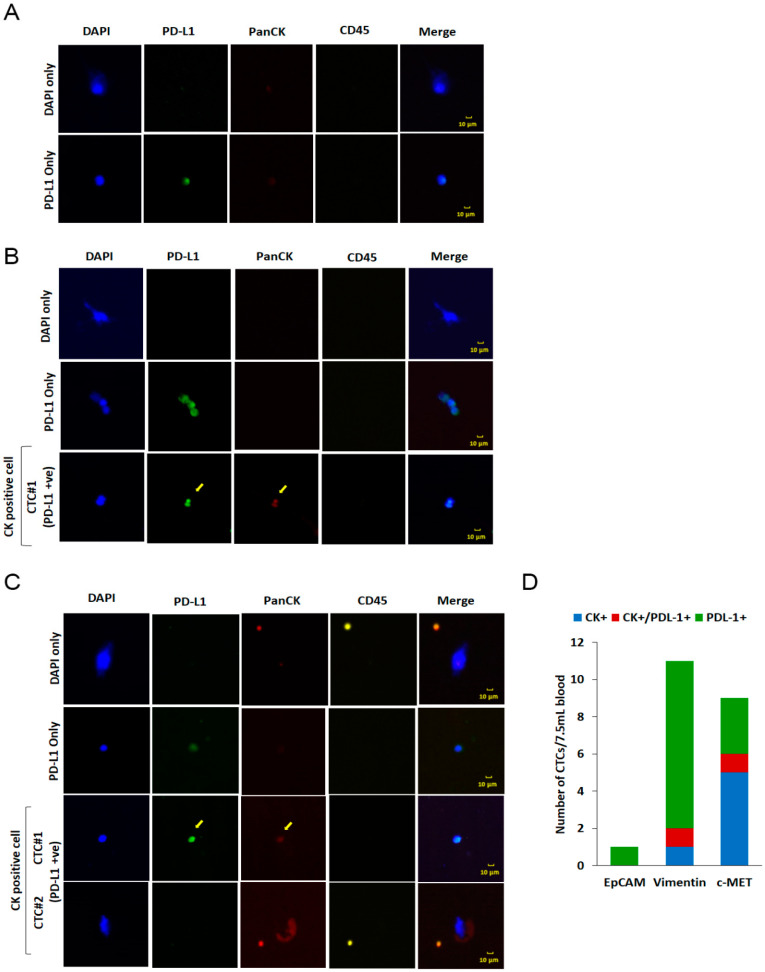
Isolation and characterization of CTCs from a cholangiocarcinoma patient: (**A**) Immunofluorescence image showing EPCAM-positive cells isolated from a cholangiocarcinoma patient. EPCAM-positive cells were immunostained with DAPI for nuclear staining (blue), an anti-PDL1 antibody (Alexa flour 488; Green), and an anti-Pan CK antibody (Alexa flour 594; Orange red) to detect CTCs; an anti-CD45 antibody was used for leukocytes (Cy3; Yellow). (**B**) Vimentin-positive cells isolated from a cholangiocarcinoma patient were immunostained with DAPI for nuclear staining (blue), an anti-PDL1 antibody (Alexa flour 488; Green), and an anti-Pan CK antibody (Alexa flour 594; Orange red) to detect CTCs; an anti-CD45 antibody was used for leukocytes (Cy3; Yellow). (**C**) MET-positive cells isolated from a cholangiocarcinoma patient were immunostained with DAPI for nuclear staining (blue), an anti-PDL1 antibody (Alexa flour 488; Green), and an anti-Pan CK antibody (Alexa flour 594; Orange red) to detect CTCs; an anti-CD45 antibody was used for leukocytes (Cy3; Yellow). The yellow arrow indicates CK+/PDL1+ cells. All microscopic images were obtained using a Nikon Eclipse Ni microscope equipped with an Infinity3 camera. (**D**) Graph showing the difference in CTC counts determined using EPCAM-, vimentin- or ME-based methods. The number of PDL1-positive CTCs observed in each case is also shown.

Cholangiocarcinoma is the second most common primary hepatic malignancy, most common in Asian countries and with an increasing worldwide incidence. Most patients are asymptomatic in the early stages of the disease, making the early diagnosis of the disease challenging [47]. Studies on the role of CTCs in providing prognostic and diagnostic information for cholangiocarcinoma are limited. Yang et al. have previously reported that CTCs are associated with poor overall survival in cholangiocarcinoma patients [48]. Moreover, recent studies on CTCs have suggested that EPCAM-based CTC detection may be insufficient, and CTC isolation and detection based on a broad range of markers might improve CTCs’ prognostic value. Here, we enumerated and analyzed CTCs from a cholangiocarcinoma patient using the epithelial marker EPCAM (Figure 5A) and the non-epithelial markers vimentin (Figure 5B) and MET (Figure 5C). As it was expected, in concordance with previous reports, CTC number varied depending on the cell surface marker employed for their isolation. While no CTCs were observed when CTCs were isolated using EPCAM, the vimentin- and MET-based methods resulted in the isolation of one and five CTCs, respectively (Figure 5D). Further studies with a larger number of patients are imperative to fully understand the role of CTCs in the prognosis and drug response of cholangiocarcinoma patients. Taken together, our results indicate that GenoCTC can efficiently isolate CTCs from different cancers and can be adapted to isolate CTCs using varying cell-surface markers. Furthermore, the establishment of prognostically relevant PDL1-positive or MET-positive CTC assays would be a promising strategy for monitoring relapse and disease progression and providing therapeutic strategies to treat cancer patients, ultimately achieving patient-specific medication through companion diagnostics.

## 4. Discussion

The effective capture of extremely low-abundant cells is the most challenging part of CTC isolation and analysis. Immunomagnetic isolation and positive magnetophoresis based on labelling the target cells with magnetic particles are being widely used for the isolation of CTCs. The FDA-approved system for CTC isolation CellSearch uses an anti-EpCAM antibody-coated ferrofluid for the isolation of EpCAM-positive CTCs from whole blood. To improve the efficiency of CTC capture, microelectromagnets consisting of multiple layers of lithographically patterned wires have been successfully used [49,50,51]. The integration of wire patterns can increase the average CTC capture rate significantly when compared to that of devices without micromagnets. Our previous study and another study by Kim et.al, used a ferromagnetic wire pattern along with an external permanent magnet in a microchip to efficiently separate CTCs from peripheral blood [21,49]. The pattern design can greatly influence the capture efficiency in these microdevices. The “V”-shaped pattern prevents the cells from getting trapped in a vortex flow at the channel walls and reduces the interaction between CTCs and microchannel walls, thereby minimizing the shear stress on the cells. Technology to accurately regulate the balance between the hydrodynamic and magnetophoretic drag force and the microfluidic force, as well as precise fabrication of the device accordingly are hence crucial [52].

Here, we presented a sensitive microfluidic approach capable of isolating biomarker-positive cancer cells. The putative CTCs were isolated from whole blood on the basis of magnetophoresis with antibody-coated magnetic beads and a system using a ferromagnetic wire pattern. Under the influence of the external permanent magnet, the magnetic field near the ferromagnetic wires was altered locally and allowed for high-gradient magnetic separation. When magnetic bead-bound CTCs passed over the wire pattern, the increased magnetic force and the hydrodynamic drag force caused the CTCs to follow the wire pattern [49,51,52]. Most of the cells with a high number of beads bound on their surface became trapped in the first magnetic wire stripe. Those cells that did not move along the first wire pattern stripe, were eventually trapped as they passed along the wire pattern chip. However, it needs to be noted that the separation of the CTCs from peripheral blood largely depends on the number of magnetic beads bound to the cells, which in turn depends on the expression level of the biomarker used.

In this pilot study, we showed the suitability of GenoCTC to isolate CTCs from cancer cell-spiked samples and whole blood specimens from NSCLC patients, the latter indicating clinical feasibility. An increase in CTC count was observed almost a month prior to the clinical diagnosis of a progressive disease (Patient #3 and patient #10), indicating the clinical relevance of the isolated CTCs. Though the study was conducted in a small patient cohort to show the applicability of GenoCTC, the results showed a strikingly reasonable correlation between the number of isolated CTCs and clinical progression of the disease as well as clinical response and non-response to treatment. In addition to the enumeration of CTCs, cellular characterization of the isolated CTCs revealed that the expression of PDL1 and MET can be identified in NSCLC patients. The study of the clinicopathological role of PDL1 in cholangiocarcinoma patients is in its infancy. In this study, we showed that PDL1 expression could be observed in CTCs isolated from a cholangiocarcinoma patient. Interestingly, we also observed cells that were CK-/CD45-/PDL1+. The role of these cells in disease prognosis is unclear. With the emergence of PDL1-targeted immune-modulating therapy, the characterization of PDL1 expression status in CTCs is attracting the interest of researchers, and in-depth studies using large patient cohorts are necessary.

Immunomagnetic CTC separation methodologies mostly rely on biomarker expression, more commonly, EPCAM expression. However, the enrichment of EPCAM-positive CTCs has raised controversies because CTCs that have undergone EMT are generally overlooked [53,54,55]. Many studies have reported that EPCAM is still the most clinically relevant CTC biomarker. EPCAM overexpression in cancer cells plays a critical role in cancer cell migration, proliferation, and differentiation, consequently imparting the potential to metastasize to cancer cells, after extravasation [56,57,58]. Wit et al. explored both EPCAM-positive and -negative CTCs in metastatic lung cancer patients and found that the presence of EPCAM-positive CTCs was correlated with poor outcome, whereas there was no significant correlation between the presence of EPCAM-negative CTCs and overall survival [59]. Schulze et al. also described the detection of EPCAM-positive CTCs in hepatocellular carcinoma patients (HCC) and found that these cells were detected more frequently in patients with intermediate or advanced HCC than in those with local limited disease; accordingly, their presence was associated with poor overall survival [60].

Recently, several new technologies including label-free size-based enrichment, antibody-coated microposts in microfluidic chips [61] or herringbone channels [13,62], etc. have been developed to isolate putative CTCs. Label-free methods to isolate large CTCs still present unresolved challenges in that they miss small or less stiff CTCs and allow lower purity than other technologies because of leukocyte contamination [63,64]. Accordingly, these methods may provide unreliable clinical information when CTCs genome is characterized; therefore, they may not be the best solution to isolate CTCs [7,18]. On-chip capture methods, like those based on antibody-coated chips or herringbone channels, utilize molecular properties and take advantage of the 3D structure of the channels to increase the surface area available for coating with the antibody or aptamer of choice [65]. These technologies, although promising, present inherent constraints in the large-scale production of chips, which require detailed surface chemistry modifications. Herringbone chips, with their simplified architecture, are more appropriate for large-scale production; however, the loss of cells and the damage-induced changes during the release of the cells from the chip for downstream molecular or single-cell genomic assays also pose several challenges. A simple, high-purity CTC enrichment system suitable for mass-production and appropriate for large multi-center studies to validate the clinical relevance of CTCs is still in demand.

## 5. Conclusions

Here, we showed that GenoCTC can be easily used with different cell surface biomarkers including vimentin and MET, as shown in Figure 5. Vimentin-positive CTCs could be isolated to gain a deep understanding of the molecular status of CTCs. Studies are further required to examine the performance of the GenoCTC system using a multiple-biomarker cocktail to isolate a wide range of CTCs simultaneously. Moreover, the investigation of clinically relevant prognostic markers on CTCs can be a valuable tool in enabling companion diagnostics to design the most suitable treatment strategy to improve patients’ outcomes. The isolation of rare CTCs from whole blood, without any pre-processing of the samples, could be achieved using the GenoCTC device. Spiking experiments with MCF7 cells showed that up to 6 cells in 1 mL of blood could be recovered without compromising the purity of the recovered cells, with approximately a 70% recovery rate. This study demonstrated a proof of concept, showing the applicability of GenoCTC and confirming the potential of our system in isolating CTCs. We are also currently developing a fully automated GenoCTC, with improved selectivity and specificity for the targeted cells, no cross-contamination between samples, and improved purity, which will allow the isolation of CTCs for further single-cell analyses or genomic/epigenomic research. The current study is limited due to the small patient cohort that was investigated. The patients also varied for the treatment received. Further studies with larger cohorts are imperative to obtain clinically reliable data as well as to get further insight into the role of the molecular status of CTCs on disease progression, prognosis, and recurrence. Ongoing studies are currently investigating large cohorts of various cancer patients over time, using the fully automated GenoCTC, to highlight the applicability of GenoCTC as a companion diagnostic.

## 6. Patents

GenoBio Corp. holds patents for the disposable separation chip and cell separation system and a non-exclusive license from Electronics and Telecommunications Research Institute, Daejeon, Republic of Korea, for the development and manufacture of GenoCTC-chip.

## Supplementary Materials

The following are available online at https://www.mdpi.com/2072-666X/11/6/560/s1, Figure S1: Recovery and separation rate of MCF7 cells; Figure S2: EpCAM expression in epithelial cancer cell lines as analysed by flow cytometry Figure S3: Recovery rate and separation rate of MCF7 spiked to whole blood; Video S1: Separation of EpCAM-positive CTCs from whole blood of NSCLC patients; Video S2: Separation of EpCAM-positive MCF7 cells spiked into whole blood of healthy individual.
