# Supplementary material for: An Immune–Magnetophoretic Device for the Selective and Precise Enrichment of Circulating Tumor Cells from Whole Blood
Source: Micromachines (Basel). 2020 May 30;11(6):560. doi: 10.3390/mi11060560 (PMC7345362; doi:10.3390/mi11060560)
Supplement: Supplementary file 1 [file micromachines-11-00560-s001.zip › micromachines-805701-supplementary materials-for final.docx]

Supplementary Materials

An Immune-Magnetophoretic Device for the Selective and Precise Enrichment of Circulating Tumor Cells from Whole Blood

Chaithanya Chelakkot, Jiyeon Ryu, Miyoung Kim, Jin-Soo Kim, Dohyeong Kim,
Juhyun Hwang, Sung Hoon Park, Seok Bum Ko, Jeong Won Park, Moon Youn Jung, Ryong Nam Kim, Kyoung Song, Yu Jin Kim, Yoon-La Choi, Hun Seok Lee
and Young Kee Shin

| 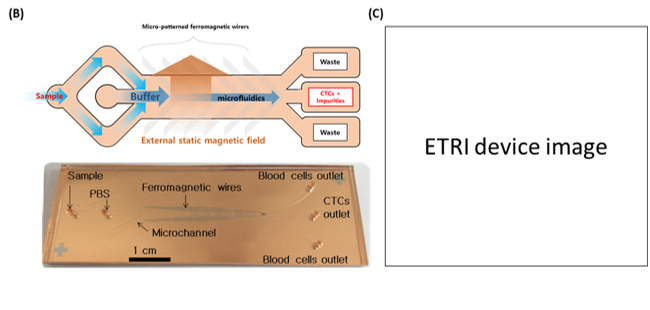 |  |
| --- | --- |
| **(A)** |  |
| 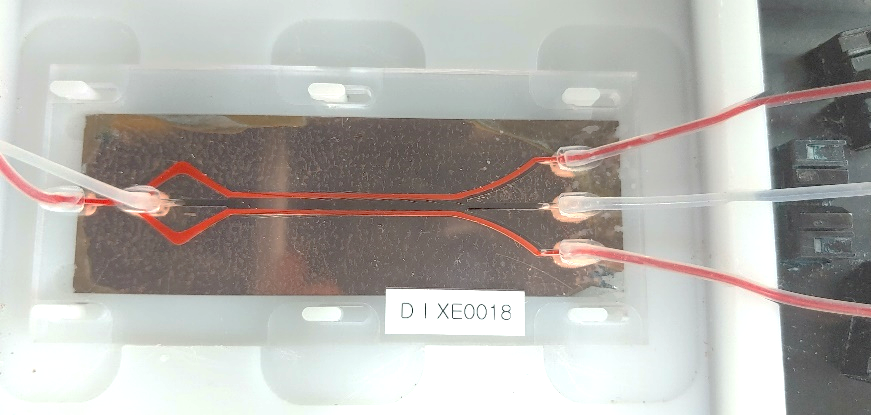 |  |
| **(B)** |  |

**Figure S1.** GenoCTC microfluidic chip: The image shows the microchip used for separation in the GenoCTC device. (**A**) The single use assembled microchip in which the ferromagnetic wire pattern glass substrate, and the upper microfluidic channel are assembled in a single unit. (**B**) The actual image of blood sample being separated in a reusable microchip.


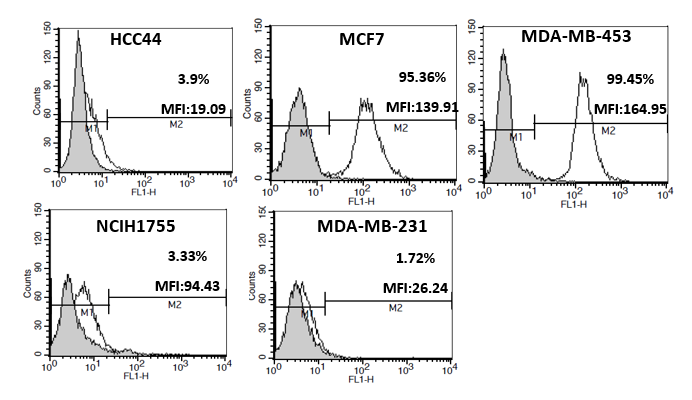


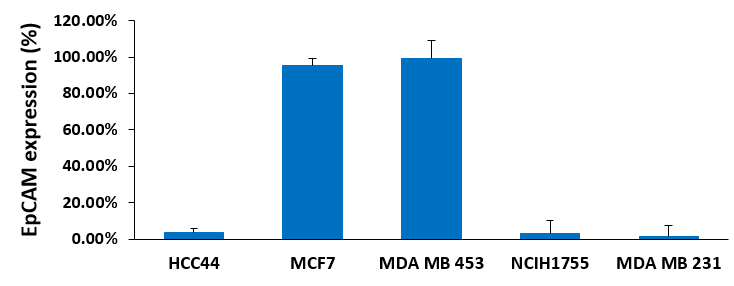


**MDA-MB-231**

**Figure S2.** EpCAM expression in epithelial cancer cell lines as analyzed by flow cytometry. The image shows the histogram curve and EpCAM expression percentage in the 5 cell lines, as analyzed by flow cytometry.

**Figure S3:** Recovery and Separation rate of MCF7 cells spiked to whole blood: The graph shows the recovery and separation rate of MCF 7 cells spiked to whole blood from a healthy donor, after processing in the GenoCTC device. A high recovery and separation rate of approximately 80% was observed when 200 cells were spiked to the whole blood. The recovery and separation rate did not show a significant difference when as low as 12 cells were spiked. The recovery and separation rate dropped slightly when 6 cells were spiked and drastically when less than 6 cells were spiked. A recovery and separation rate of approximately 40% was observed when 3 cells were used for the spiking test. .

Supplementary Video S1

The video shows the separation of EPCAM positive CTCs from the whole blood of an NSCLC patient. The microfluidic channel, the ferromagnetic wire, and the blood flow can be seen. At 8 second time point, the passage of a CTC to the sample collecting channel can be seen.

Supplementary Video S2

The video shows the separation of EPCAM positive MCF7 cells spiked into whole blood. 2000 cells were spiked in 5 mL of whole blood and separated using the GenoCTC device. The cells can be seen following the wire pattern and getting collected in the center lane.
